# A Prognostic Role for Circulating microRNAs Involved in Macrophage Polarization in Advanced Non-Small Cell Lung Cancer

**DOI:** 10.3390/cells10081988

**Published:** 2021-08-05

**Authors:** Alexia Monastirioti, Chara Papadaki, Konstantinos Rounis, Despoina Kalapanida, Dimitrios Mavroudis, Sofia Agelaki

**Affiliations:** 1 Laboratory of Translational Oncology, School of Medicine, University of Crete, Vassilika Vouton, 71003 Heraklion, Crete, Greece; monasal91@gmail.com (A.M.); chapapadak@uoc.gr (C.P.); mavroudis@uoc.gr (D.M.); 2 Department of Medical Oncology, University General Hospital of Heraklion, Vassilika Vouton, 71110 Heraklion, Crete, Greece; rounis@gmail.com (K.R.); despoinakalapanida87@gmail.com (D.K.)

**Keywords:** circulating miRNAs, NSCLC, platinum-based chemotherapy, immune response, survival, tumor associated macrophages, TAMs, tumor microenvironment, TME

## Abstract

Circulating microRNAs (miRNAs) are key regulators of the crosstalk between tumor cells and immune response. In the present study, miRNAs (let-7c, miR-26a, miR-30d, miR-98, miR-195, miR-202) reported to be involved in the polarization of macrophages were examined for associations with the outcomes of non-small cell lung cancer (NSCLC) patients (N = 125) treated with first-line platinum-based chemotherapy. RT-qPCR was used to analyze miRNA expression levels in the plasma of patients prior to treatment. In our results, disease progression was correlated with high miR-202 expression (HR: 2.335; *p* = 0.040). Additionally, high miR-202 expression was characterized as an independent prognostic factor for shorter progression-free survival (PFS, HR: 1.564; *p* = 0.021) and overall survival (OS, HR: 1.558; *p* = 0.024). Moreover, high miR-202 independently predicted shorter OS (HR: 1.989; *p* = 0.008) in the non-squamous (non-SqCC) subgroup, and high miR-26a was correlated with shorter OS in the squamous (SqCC) subgroup (10.07 vs. 13.53 months, *p* = 0.033). The results of the present study propose that the expression levels of circulating miRNAs involved in macrophage polarization are correlated with survival measures in NSCLC patients, and their role as potential biomarkers merits further investigation.

## 1. Introduction

Even though the discovery of targeted drugs [1] and immunotherapy [2] brought major changes in the field of precision medicine in NSCLC [2,3], high cost, the detection of targetable alterations (e.g., EGFR, c-MET, ALK, ROS1 and BRAF) [4] in less than 30% of cancer patients and the efficacy of immunotherapy in only a subset of patients, limit the number of patients who can benefit. As a result, despite recent progress, platinum-based chemotherapy, alone or in combination with immunotherapy, remains the cornerstone of treatment for a significant number of advanced or metastatic NSCLC patients with no targetable genetic alterations [5]. Furthermore, chemotherapy is recommended following failure of targeted therapies or immunotherapy, and in patients with early stages of the disease.

Platinum compounds bind to DNA and form platinum-DNA adducts, which interfere with transcription and DNA replication, ultimately resulting in programmed cell death [6]. In response to cisplatin (CDDP), cells activate multiple repair pathways, such as homologous recombination, nucleotide excision repair, and Fanconi anemia, to detect and repair DNA adducts. The ability of cells to repair DNA crosslinks is considered a critical determinant for the cytotoxic effects of CDDP, and mutations or deregulated expression of genes involved in DNA repair pathways is associated with sensitivity to platinum agents [7]. Nevertheless, CDDP sensitivity is not always associated with a defective DNA damage response, and the quest for markers predictive of outcomes with platinum-based treatment remains a significant challenge.

Besides the formation of DNA cross-links, immunomodulatory effects have also been ascribed to platinum compounds. Collectively, CDDP positively regulates MHC class I expression, stimulates the recruitment and proliferation of effector T cells and antigen-presenting cells (APCs), enhances cytotoxic effector T-cell activity and counteracts immunosuppressive factors within the tumor, thereby creating an inflammatory tumor microenvironment (TME) [8]. Intense lymphocytic infiltration, suggestive of an existing anti-tumor immune response within the TME, was shown to be a favorable prognostic marker for survival in resected NSCLC; however, there are no robust data on the prognostic significance of different subsets of immune cells [9].

Macrophages are crucial components of the innate immune response; and tumor associated macrophages (TAMs) participate in the regulation of tumor growth, survival and the anti-tumor adaptive immune response [10]. Macrophages demonstrate a high level of plasticity, having the ability to transit between M1 and M2 polarized phenotypes. Macrophage polarization has prognostic value for various tumor types. The M1-like phenotype promotes anti-tumor responses, and the M2-like phenotype exhibits tumor-supporting functions [11,12,13]. The TME of NSCLC contains one of the highest TAM densities among human cancers [14]. Several studies have reported associations of their presence with patient outcomes [15]. A higher M1-like phenotype content in NSCLC has been linked to prolonged survival of patients, whereas M2-polarization of macrophages has been associated with adverse patient outcomes [16,17,18].

MiRNAs, a class of small non-coding RNAs, suppress gene expression at the post-transcriptional level [19], acting either as oncomirs or as tumor suppressors depending on the tumor type [19], and a variety of studies have demonstrated that miRNAs may represent potential biomarkers in NSCLC patients treated with platinum-based chemotherapy [20,21]. MiRNAs have been reported to regulate immune system development and function, and deregulated expression of several of these miRNAs can lead to hematological cancers [22]. Furthermore, miRNAs are central mediators of the crosstalk between tumor cells and the immune system [23]; and they have been reported to participate in the regulation of macrophage production and reprogramming, and to modulate their polarization [10,24,25].

The miRNAs let-7c, miR-26a and miR-202 have been found to induce macrophage polarization towards the M2-like phenotype [25,26,27]. Tumor-originated miR-30d was shown to increase the expression and secretion of IL-10 that promotes M2-like phenotype polarization [28]. On the contrary, miR-98 targets IL-10 and suppresses M2-like phenotype polarization [25]. Finally, in experimental models of colorectal cancer, miR-195 has been proposed to inhibit M2-like phenotype polarization and to restrict tumor growth [29].

Tissue-based analyses cannot capture the dynamic nature of the tumor–host interactions, in contrast to circulating markers, which may conclude the systemic response to the tumor, providing, in parallel, the opportunity for repeated monitoring. In the current study, we hypothesized that the expression levels of the aforementioned let-7c, miR-26a, miR-30d, miR-98, miR-195 and miR-202, when assessed in the plasma, may predict clinical outcomes in NSCLC patients treated with first-line platinum-based chemotherapy.

## 2. Materials and Methods

### 2.1. Patients’ Characteristics

In the current analysis, patients with advanced (not amenable to radical loco-regional treatment) or metastatic NSCLC, treated with first-line platinum-based chemotherapy at the Department of Medical Oncology, University General Hospital of Heraklion, Crete, Greece, from 2009 to 2017, with available plasma samples, obtained prior to the initiation of first-line chemotherapy, were retrieved (N = 195). Patients with plasma samples that changed color towards pink/red, suggesting that the samples were hemolyzed, were excluded from the study (N = 47). Clinical characteristics and follow-up data of the patients had been collected prospectively. Response to treatment was evaluated by computed tomography (CT) and magnetic resonance imaging (MRI) scans according to RESIST 1.1 criteria [30]. All patients signed informed consent forms before plasma sample collection.

### 2.2. Characteristics of the Healthy Blood Donors

As a control group for normalization of the miRNAs’ expression values, blood samples from healthy volunteers (N = 33) were used. Samples from volunteers were obtained as part of the volunteer blood donation procedure in the Blood Bank Department of the University General Hospital of Heraklion, Crete, Greece. The median age of this group was 63 years, of whom 30 were males and 3 were females. All volunteers signed informed consent forms for their participation in the current research program.

### 2.3. Blood Sample Collection

Peripheral blood from patients and healthy volunteers was collected in ethylenediaminetetraacetic acid (EDTA) tubes. Within 2 h, plasma was isolated from whole blood in a two-step centrifugation, firstly in 1300× *g* (15 min, 4 °C) and secondly in 2000× *g* (15 min, 4 °C, cellular debris removal). Blood samples of patients and healthy volunteers were stored at −80 °C until further use.

### 2.4. RNA Isolation from Plasma Samples

Total RNA was extracted from 400 μL of plasma using Trizol-LS (Ambion, Life Technologies, Carlsbad, CA, USA) according to manufacturer’s instructions. After denaturation, 5 μL containing 25 fmoles of the synthetic miRNA from C. elegans, cel-miR-39 (Qiagen Inc., Germantown, MD, USA), and 300 μL of chloroform, were added to each sample. *Cel*-miR-39 was used as an exogenous control to allow for normalization of sample-to-sample variations and chloroform for the separation of the aqueous phase from the organic. Following incubation and centrifugation, the aqueous phase was transferred to an eppendorf tube, where equal volumes of isopropanol plus 1 μL of glycogen (13 µg/mL total, QIAGEN, GmbH, Hilden, Germany) were added to the sample for RNA precipitation. The samples were incubated overnight at −80 °C and RNA pellets were washed with 75% ethanol (Et-OH), air-dried and finally resuspended in 50 μL RNAse-free water. Total RNA from all samples was kept at −80 °C until further use in the subsequent real-time PCR [6].

### 2.5. Quantitative Real-Time PCR Analysis and miRNA Expression

Reverse transcription and RT-qPCR were performed according to manufacturer’s instructions. Total RNA input of 1.2 μL was reverse transcribed using the TaqMan miRNA Reverse Transcription kit and miRNA-specific stem-loop primers (assays ID for each miRNA are provided in Table S1, Applied Biosystems, Foster City, CA, USA). The reverse transcription reaction was performed in a final volume of 5 μL, containing 1 mM dNTPs, 0.5× PCR Reverse Transcription Buffer, 0.5× RT-specific stem-loop primers, 16.5 units of Multiscribe Reverse Transcriptase and 1.26 units of RNase Inhibitors. The reaction was performed in a Peltier Thermal Cycler PTC-200 at 16 °C for 30 min, 42 °C for 30 min and 85 °C for 5 min. Complementary DNA (cDNA) was diluted to a 20 μL final volume, and each miRNA was assessed by RT-qPCR in a 5 μL reaction comprising of 2.5 μL Universal PCR Master Mix, 0.25 μL TaqMan miRNA Assay and 2.25 μL diluted cDNA. The RT-qPCR reaction was carried out at 95 °C for 10 min, followed by 40 cycles of 95 °C for 15 min and 60 °C for 1 min, in a ViiA 7 Real-Time PCR System (Applied Biosystems, Foster City, CA, USA). All the assays were performed in triplicates. Appropriate negative controls were used in both cDNA synthesis and RT-qPCR reactions, wherein RNA input was replaced by H_2_O and no template control was used, respectively [6]. The average expression level for each miRNA was calculated by the 2^−ΔCt^ method relative to the average of U6 snRNA.

U6 snRNA was chosen as a suitable reference gene for normalization, due to expression stability and reproducibility among the group of patients and the group of healthy volunteers (Mann–Whitney test, *p* = 0.272) (Figure S1). Acceptable mean cycle threshold (Ct) ranges were for U6 snRNA, 30 < Ct U6 < 33, and for cel-miR-39, 20 < Ct cel-miR-39 < 22. Samples with mean Ct values outside of these ranges were excluded from the analysis (N = 14 for U6 snRNA and N = 9 for cel-miR-39). The fold changes in target miRNAs relative to their expression in healthy volunteers were determined by the2^−ΔΔCt^ method [31]. Median Ct values, SD and median miRNA expression values for both patients and healthy volunteers are depicted in Table S2.

### 2.6. Statistical Analysis

Statistical analysis was performed via SPSS software package version 22.0 (statistical package of the social sciences, SPSS Inc. Chicago, IL, USA). Normality tests (Kolmogorov–Smirnov and Shapiro–Wilk test) revealed that the miRNAs relative expression values do not follow a normal distribution (*p* < 0.05). Each patient was categorized as having high or low expression according to the median value of each miRNA. More specifically, miRNA expression values higher than or equal to median values characterized patients as “high expression,” whereas those with expression values lower than the median values were characterized as “low expression.” The correlation between miRNA expression and clinicopathological characteristics was assessed by chi-squared test. The association of miRNA expression levels with disease stabilization rates (PR (partial response) or SD (stable disease) vs. PD (progressive disease)) was examined using chi-square test and the probability of developing disease progression as the best response to treatment was evaluated by applying binary logistic regression. Plasma miRNA expression levels and their correlations with PFS and OS were assessed via Kaplan–Meier method, log rank test (Mantel–Cox) and the Cox proportional hazard regression models. Multivariate Cox regression analysis was composed of parameters that had achieved statistical significance in the univariate analysis. PFS and OS were estimated from the start of first-line treatment until the date of the first documented instance disease progression and death, respectively. If a patient had not progressed or was alive at the time of data analysis, he/she was censored at the time of the last follow-up. Statistical significance was set to *p* < 0.05 (two-sided test). This report was written based on the Reporting Recommendations for Tumor Marker Prognostic Studies (REMARK) criteria [32].

### 2.7. KM Plotter Analysis

After an extensive literature review, we identified candidate miRNAs with reported roles in macrophage polarization in tumors and/or inflammation (Table S3). We then employed KM plotter database, an online tool (http://kmplot.com/analysis/, accessed on 17 March 2020), to assess the correlations between the expression of these miRNAs and overall survival in lung cancer, and to draw preliminary conclusions regarding the roles of the miRNAs in prognoses for NSCLC. KM plotter utilizes genome-wide microarray datasets that have been published over the years, and integrates a large-scale database comprising gene expression information and clinical outcome parameters of various types of cancer, suitable for the in-silico validation of biomarker candidates [33]. KM plotter analysis was performed to acquire KM survival plots, and the hazard ratios (HR), 95% confidence intervals (CI) and log-rank *p*-values were determined. When the *p*-value was <0.05, the difference was regarded as statistically significant. Let-7c, miR-26a, miR-30d, miR-98, miR-195 and miR-202 were selected based on their suggested prognostic value according to the KM plotter analysis (Figure S6), and the limited, or no, data regarding their prognostic roles as circulating biomarkers in NSCLC.

## 3. Results

### 3.1. Patients’ Characteristics and Study Design

**Figure 1:**
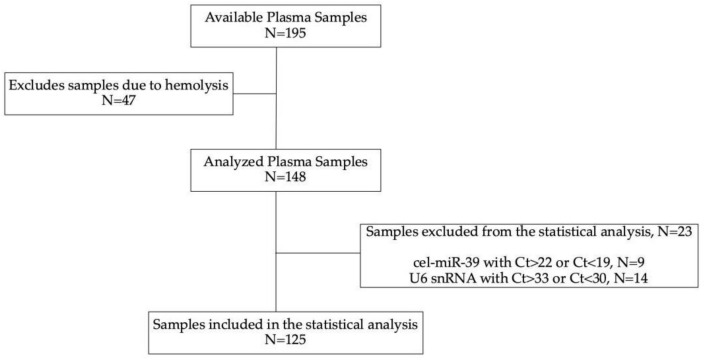
Flow of the study, schematic illustration.

Patients’ characteristics are depicted in Table 1, and the flow of the study is depicted in Figure 1. The median age was 65 years (range: 37–88); 86.4% of the patients were male; 68% of the patients had non-SqCC histologic type; 26.4% of the patients experienced PR, 39.2% SD and 34.4% PD.

### 3.2. miRNA Expression and Clinicopathological Characteristics

Out of patients with ≥3 metastatic sites, 69.8% had low miR-26a expression, in contrast to 30.4% of patients with high expression (Mann–Whitney U test, *p* = 0.030) (Figure S2). Additionally, 77.8% of patients with low miR-26a developed brain metastases, compared to 22.2% of patients with high miR-26a expression (Mann–Whitney test, *p* = 0.008) (Figure S2). Finally, 21.1% of patients with high and 78.9% of patients with low let-7c expression developed bone metastases (chi-square test, *p* = 0.015). No other correlations were observed when comparing miRNA expression with patients’ clinicopathological characteristics.

### 3.3. miRNA Expression and Their Effect on Response to Treatment

Univariate binary logistic regression (N = 125) analysis revealed that only high miR-202 expression (HR: 2.335, 95% CI: 1.038–5.254; *p* = 0.040) was correlated with the probability of developing progressive disease as the response to chemotherapy (Table 2). Specifically, out of patients with high miR-202 expression, 64.9% developed PD, compared to 35.1% of patients with low miR-202 (chi-square test, *p* = 0.030). However, multivariate binary logistic regression analysis for responses was not feasible due to the lack of other statistically significant factors.

### 3.4. miRNA Expression and Association with Survival Outcomes

**Figure 2:**
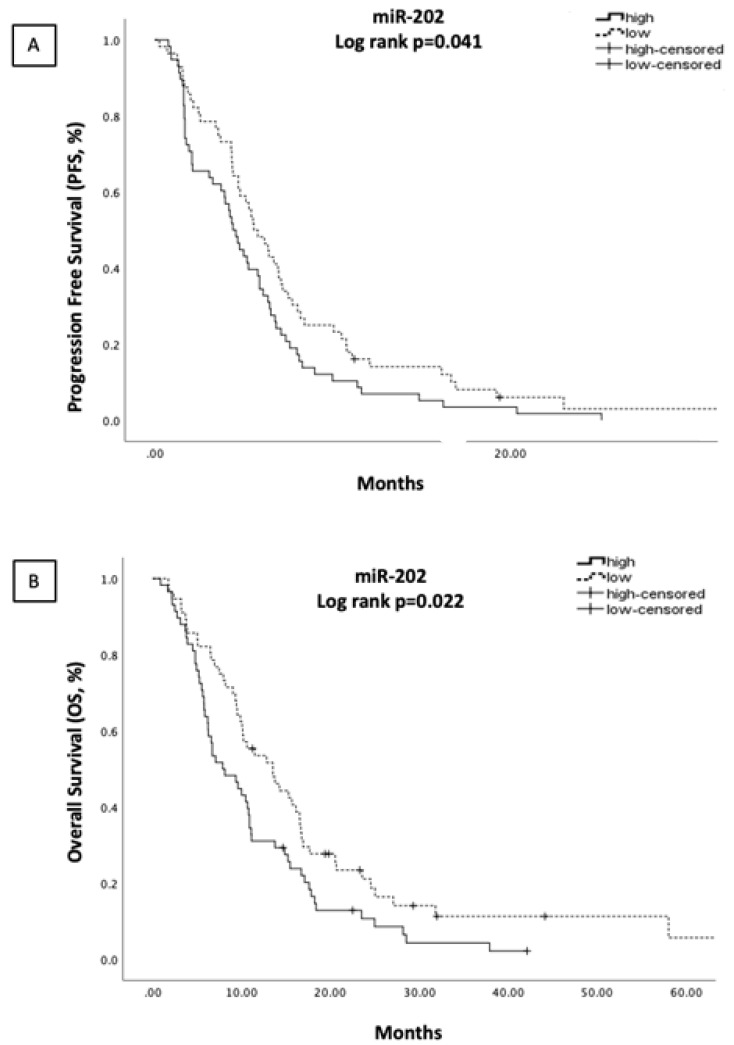
Kaplan-Meier analysis for PFS (**A**) and OS (**B**) according to miR-202 expression levels in the plasma of NSCLC patients (N = 125). Median expression values classified patients into high and low expression groups. Curves were compared using the log rank test. *p* values are shown.

The median PFS was 5.13 months (range: 0.27–102.0 months) and the median OS was 10.20 months (range: 0.90–102.0 months) in the whole group of patients (N = 125). Patients with high miR-202 expression had shorter PFS and shorter OS (4.4 vs. 6.17 months, *p* = 0.041; and 7.87 vs. 13.53 months, *p* = 0.022) (Figure 2A,B, respectively). The expression levels of the remaining miRNAs were not associated with either PFS or OS (Figure S3).

Univariate cox regression analysis (N = 125) revealed that high miR-202 was associated with shorter PFS (HR: 1.455, 95% CI: 1.000–2.118; *p* = 0.048) (Table 3) and shorter OS (HR: 1.596, 95% CI: 1.074–2.292; *p* = 0.020) (Table 4). Other factors associated with shorter PFS were male gender (HR: 1.831, 95% CI: 1.089–3.078; *p* = 0.023), the presence of ≥3 metastatic sites (HR: 2.014, 95% CI: 1.301–3.118; *p* = 0.002) and the presence of liver metastases (HR: 2.192, 95% CI: 1.413–3.402; *p* < 0.001) (Table 3). PS ≥ 2 (HR: 2.289, 95% CI: 1.353–3.871; *p* = 0.002), the presence of ≥3 metastatic sites (HR: 1.589, 95% CI: 1.028–2.456; *p* = 0.037) and the presence liver metastases (HR: 1.826, 95% CI: 1.190–2.802; *p* = 0.006) were associated with shorter OS (Table 4).

In multivariate cox regression analysis (N = 125), high miR-202 expression emerged as an independent prognostic factor for both worse PFS and worse OS (HR: 1.564, 95% CI: 1.068–2.289, *p* = 0.021, Table 3; and HR: 1.558, 95% CI: 1.060–2.291, *p* = 0.024, Table 4). Male gender (HR: 2.232, 95% CI: 1.262–3.946; *p* = 0.006) and liver metastasis (HR: 1.877, 95% CI: 1.139–3.094; *p* = 0.014) were independent predictors for poor PFS, (Table 3), whereas PS ≥ 2 (HR: 2.065, 95%CI: 1.215–3.581; *p* = 0.008) and liver metastases (HR: 1.666, 95% CI: 1.033–2.687; *p* = 0.036) independently predicted poor OS (Table 4).

### 3.5. Correlations of Clinicopathological Characteristics and miRNA Expression with Patient Outcomes According to Histologic Subtype

Patients were classified into the SqCC (Ν = 40) and non-SqCC (Ν = 85) subgroups based on their histologic subtypes. The characteristics for each group of patients are summarized in Table 1. No association was observed between miRNA expression levels and histologic subtype. In addition, there was no statistically significant correlation of miRNA expression with response to treatment based on the histologic subgroup.

**Figure 3:**
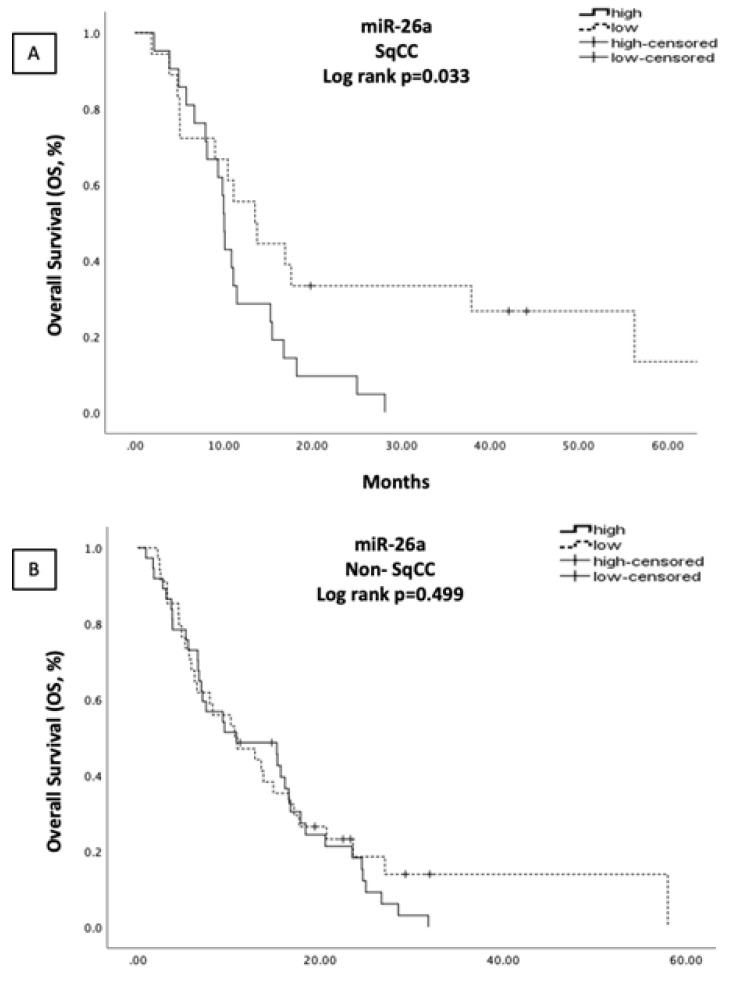
Kaplan–Meier analysis for OS based on miR-26a expression levels in the plasma of SqCC (**A**, N = 40) and non-SqCC (**B**, N = 85) NSCLC patients. Median expression values classified patients into high and low expression groups. Curves were compared using the log rank test; *p*-values are shown.

In the SqCC subgroup, no correlations were found regarding miRNA expression and clinicopathological characteristics. Moreover, no associations were revealed between miRNA expressions and PFS. Patients with high miR-26a had shorter OS compared to patients with low expression (10.07 vs. 13.53 months, *p* = 0.033) (Figure 3) and in Univariate Cox regression analysis (N = 40), high miR-26a expression (*p* = 0.047) was correlated with poor OS, along with PS ≥ 2 (*p* = 0.048) (Table S4). However, none of the aforementioned factors was revealed to be independent in multivariate Cox regression analysis (Table S4).

**Figure 4:**
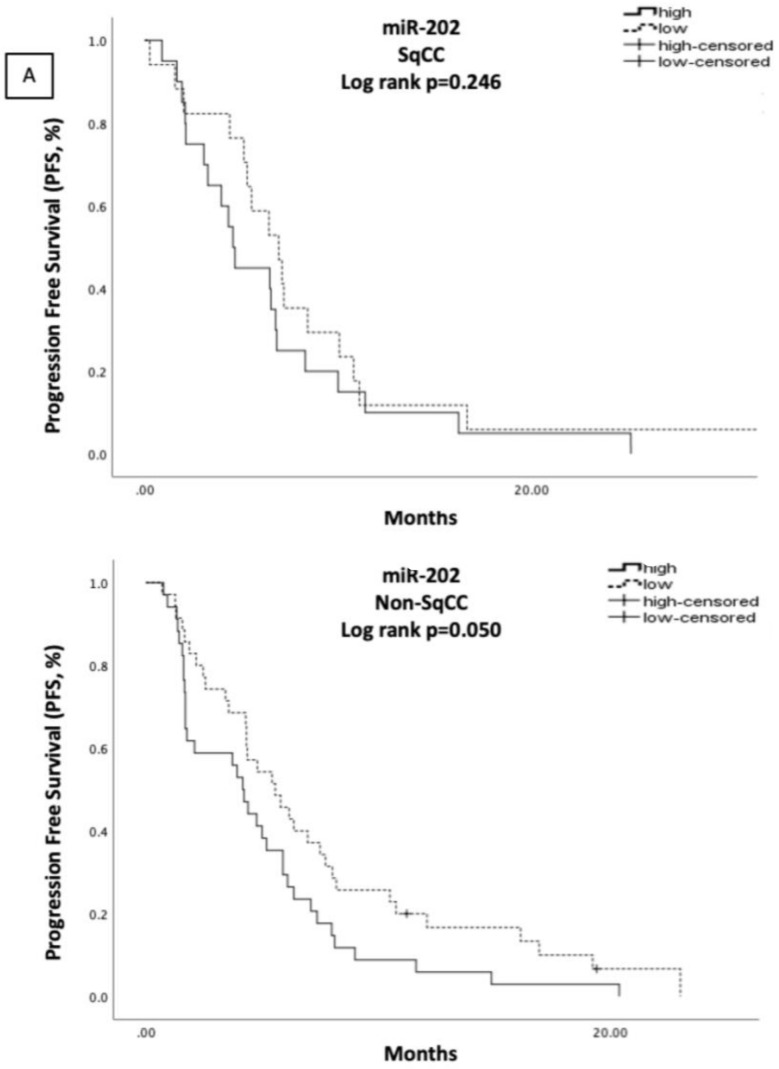

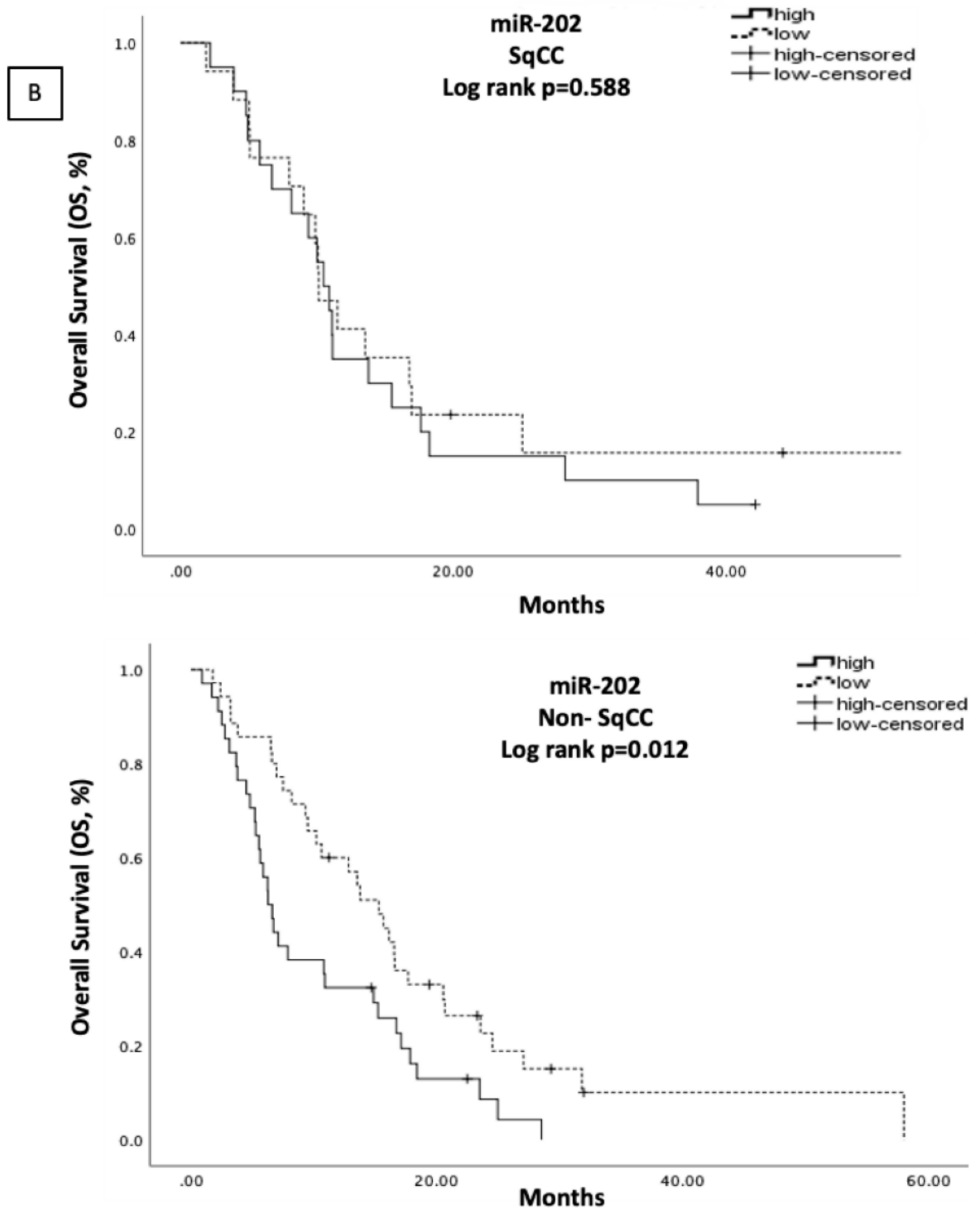
Kaplan–Meier analysis for PFS (**A**) and OS (**B**) according to miR-202 expression levels in the plasma of SqCC (N = 40) and non-SqCC (N = 85) NSCLC patients. Median expression values classified patients into high and low expression groups. Curves were compared using the log rank test. *p* values are shown.

In the non-SqCC subgroup, low miR-26 was associated with the presence of brain metastases (78.6% vs. 21.4%, low vs. high, respectively; *p* = 0.011). No other correlations were observed when comparing miRNA expression and clinicopathological characteristics in this patient subset. Patients with high miR-202 expression had shorter median PFS and OS compared to those with low miR-202 expression (4.17 vs. 5.80 months, *p* = 0.050; and 6.27 vs. 15.30 months, *p* = 0.012) (Figure 4A,B, respectively). The remaining miRNAs had no statistically significant associations with survival measures (Figure S4 and S5). High miR-202 expression (HR: 1.989, 95% CI: 1.196–3.309; *p* = 0.008), however, emerged as the only independent prognostic factor for worse OS in the non-SqCC subgroup (Table S5).

## 4. Discussion

Circulating miRNAs have been recognized as potential prognostic biomarkers in cancer patients [34]. In the present study, the expression levels of let-7c, miR-26a, miR-30d, miR-98, miR-195 and miR-202 were assessed in the plasma of NSCLC patients treated with first-line platinum-based chemotherapy and evaluated regarding clinical outcomes. These miRNAs were selected according to their reported roles in macrophage polarization [25,26,27,28,29]. Collectively, in our results, high miR-202 expression was associated with disease progression. Moreover, high miR-202 was revealed as an independent prognostic factor for shorter PFS and OS in the whole group of patients, and in the non-SqCC subgroup. In the SqCC subgroup, only high miR-26a expression was correlated with shorter OS.

MiR-202 has been implicated in the regulation of the macrophage response to bacterial infection [35,36,37]. Furthermore, miR-202 has been reported to inhibit the immune suppressor signal transducer and activator of transcription 3 (STAT3) [38], whose activation in the TME has been associated with M2-like phenotype and poor patient prognosis [39]. MiR-202 belongs to the let-7 family and has been identified as a tumor suppressor in many cancer types, including NSCLC [38,40,41,42], where it has been also reported to enhance cisplatin efficacy through Ras/MAPK targeting [43]. Reduced miR-202 expression levels have been demonstrated in lung cancer tissues [38,42]; low miR-202 expression levels are associated with tumor stage and lymph node metastasis [38].

In contrast to the reported tumor-suppressing activity of miR-202 in lung cancer, our results, for the first time, report that high plasma miR-202 expression independently predicted shorter survival in the whole group of patients and in the non-SqCC subgroup. In general, there is limited evidence regarding the associations of circulating miR-202 with clinical outcomes in cancer patients. In accordance with our results, circulating mir-202 expression was increased in breast cancer patients compared to healthy individuals [44] and was associated with tumor aggressiveness and shorter survival [45]. Our data also imply that circulating miR-202 may have differential prognostic implications in relation to the NSCLC histologic subtype. Interestingly, miR-202 was included in a 6-miRNA signature derived from lung tissue samples that were differentially expressed between lung adenocarcinoma and squamous carcinoma [46].

Contrary to our results, in KM plotter analysis, low tissue miR-202 expression was associated with poor survival in both SqCC and non-SqCC NSCLC (Figure S6). The inconsistency between our results and the results from KM plotter analysis could be related to the different types of samples tested (plasma versus tissue) and/or the different disease stages of patients evaluated. Specifically, KM plotter analysis was conducted using data from NSCLC patients, most of whom had early disease.

MiR-26a promotes M2-like phenotype polarization by repressing a variety of genes related to NF-κB and MAPK signaling pathways [27]. In hepatocellular carcinoma, miR-26a was shown to suppress the recruitment of macrophages in the TME [47]. MiR-26a promotes the metastatic potential of lung cancer via the modulation of metastasis-related gene expression [48], whereas in other reports, low miR-26a was associated with CDDP resistance in NSCLC cell lines [49].

In our results, high plasma miR-26a expression was associated with shorter survival in the SqCC subgroup only. To the best of our knowledge, there are no reports on the prognostic value of miR-26a in lung cancer. KM plotter analysis (Figure S6) demonstrated that, in contrast to our results in the plasma, low tissue miR-26a expression was associated with shorter OS in both SqCC and non-SqCC NSCLC (Figure S6). In general, contrasting results are reported regarding the role of circulating miRNA-26. In detail, patients with glioblastoma had significantly up-regulated serum miR-26a expression levels compared to controls [50], whereas patients with gastric cancer had decreased plasma and tissue levels compared to controls [51]. In another study, low miR-26a expression in patients with gastric cancer was associated with significantly shorter survival [52].

The biological functions of circulating miRNAs have not been clarified yet; however, they are considered to participate in extracellular cell communication processes regulating biological functions [34]. Both miR-202 and miR-26a are involved in the STAT3 signaling pathway in NSCLC [38,53]. STAT3 is frequently activated in NSCLC, has been linked to macrophage polarization balance [54] and has a pivotal role in driving tumor-promoting inflammation and the evasion of anti-tumor immunity [55]. Thus, STAT3 may provide a link between both miR-202 and miR-26a and NSCLC tumor progression. However, it should be noted here that the origin of circulating miRNAs is debatable. They could be either derived from the tumor, thereby carrying information regarding the place of origin [56], or secreted by blood cells. To further understand their roles, investigations of their associations with their respective tissue-based counterparts and/or the macrophage content or polarization in patient samples should be pursued.

In the present study, pre-analytical and analytical parameters were considered thoroughly, taking into account the variables that could lead to bias in miRNA quantification [57,58]. This study is the first to demonstrate that circulating miR-202 and miR-26a may predict treatment outcomes in NSCLC. Limitations of our study include the retrospective, exploratory nature of our analysis, and the small sample size precluding firm statistical correlations. The origins of the analyzed miRNAs were not investigated, and we cannot comment on the prognostic or predictive value of our findings. Finally, the results lack validation in an independent patient cohort.

## 5. Conclusions

In summary, our results show that the expression levels of miRNAs with reported roles in macrophage polarization, when assessed in the plasma, are associated with survival measures in patients with NSCLC treated with platinum-based chemotherapy. Further studies are necessary to clarify the origins of miR-202 and miR-26a and to confirm their role as circulating biomarkers in NSCLC.

## Figures and Tables

**Table 1:** Patients’ clinicopathological characteristics.

	All Patients		SqCC		Non-SqCC		
Characteristic	*N*	%	*N*	%	*N*	%	*p* value
Number of patients	125		40	32.0	85	68.0	
Gender							0.008 ^a^
Male	108	86.4	40	100.0	68	63.0	
Female	17	13.6	0	0.0	17	37.0	
Age (years)							0.339 ^a^
median (range)	65 (37–88)		66.5 (46–88)		63.2 (37–82)		
ECOG PS							0.354 ^a^
0	31	24.8	11	27.5	20	23.5	
1	77	61.6	22	55.0	55	64.7	
2	13	10.4	7	17.5	6	7.1	
3	4	3.2	0	0.0	4	4.7	
Stage at diagnosis							0.004 ^a^
II	1	0.8	1	2.5	0	0.0	
III	4	3.2	4	10	0	0.0	
IV	120	96	35	87.5	85	100.0	
Histology							Ns ^a^
Adenocarcinoma	77	61.6					
Squamous	40	32.0					
Others	8	6.4					
Number of metastatic sites							0.073 ^a^
0	15	12	6	15.0	9	10.6	
1	50	40	21	52.5	29	34.12	
2	33	26.4	9	22.5	24	28.23	
≥3	27	21.6	4	10.0	23	27.05	
Chemotherapy regimens							<0.001 ^a^
CDDP/TXT	46	36.8	19	47.5	27	31.76	
CDDP/GEM	33	26.4	20	50.0	13	15.3	
CDDP/PEM	44	35.2	1	2.5	43	50.59	
CDDP/other	2	1.6			2	2.35	
Response							0.715 ^a^
PR	33	26.4	13	32.5	20	23.53	
SD	49	39.2	14	35.0	35	41.17	
PD	43	34.4	13	32.5	30	35.3	

SqCC, squamous cell carcinoma; non-SqCC, non-squamous cell carcinoma; ECOG PS, Eastern Cooperative Oncology Group Performance Status; CDDP, cis-diamminedichloridoplatinum; TXT, taxotere; GEM, gemcitabine; PEM, pemetrexed; PR, partial response; SD, stable disease; PD, progressive disease; ns, non-significant; ^a^ Pearson’s chi-squared test for comparison between patients with SqCC and non-SqCC. Response to treatment was assessed according to the Response Evaluation Criteria in Solid Tumors (RECIST 1.1 criteria) [30]; number of metastatic sites depicts the number of affected organs.

**Table 2:** Binary logistic regression analysis depicting the odds ratios of the study parameters based on the probability of developing progressive disease as a response to platinum-based chemotherapy in NSCLC patients (N = 125).

	Univariate Analysis	
Binary Logistic Regression	OR (95% CI)	*p* Value
Age (<65 vs. ≥65)	1.130 (0.537–2.379)	0.747
Gender (male vs. female)	1.460 (0.513–4.158)	0.477
ECOG PS (≥2 vs. 0–1)	1.460 (0.513–4.158)	0.477
Stage at Diagnosis (IV vs. <IV)	1.333 (0.214–8.304)	0.757
Histology (SqCC vs. non-SqCC)	1.620 (0.306–8.583)	0.571
No. of Metastatic Sites (≥3 vs. 0–2)	2.209 (0.925–5.277)	0.074
Brain Metastases	1.014 (0.352–2.924)	0.979
Liver Metastases	1.877 (0.801–4.399)	0.144
Bone Metastases	1.289 (0.587–2.829)	0.527
let-7c (high vs. low)	1.201 (0.552–2.615)	0.644
miR-26a (high vs. low)	1.026 (0.476–2.211)	0.947
miR-30d (high vs. low)	1.422 (0.662–3.056)	0.366
miR-98 (high vs. low)	1.071 (0.445–2.577)	0.878
miR-195 (high vs. low)	1.047 (0.480–2.284)	0.908
miR-202 (high vs. low)	2.335 (1.038–5.254)	0.040 *

HR, hazard ratio; CI, confidence intervals; ECOG PS, Eastern Cooperative Oncology Group Performance Status; patients were classified into high or low expression groups according to the median value of each miRNA; Cox regression, * *p* < 0.05.

**Table 3:** Univariate and multivariate Cox regression analysis for progression-free survival (PFS) in NSCLC patients (N = 125).

	**Univariate Analysis**	
Cox Regression	HR (95% CI)	*p* Value
Age (<65 vs. ≥65)	1.179 (0.824–1.688)	0.368
Gender (male vs. female)	1.831 (1.089–3.078)	0.023 *
ECOG PS (≥2 vs. 0–1)	1.210 (0.724–2.025)	0.467
Stage at Diagnosis (IV vs. <IV)	1.069 (0.435–2.625)	0.884
Histology (SqCC vs. non-SqCC)	1.223 (0.826–1.812)	0.315
No. of Metastatic Sites (≥3 vs. 0–2)	2.014 (1.301–3.118)	0.002 *
Brain Metastases (yes vs. no)	1.226 (0.742–2.028)	0.426
Liver Metastases (yes vs. no)	2.192 (1.413–3.402)	<0.001 *
Bone Metastases (yes vs. no)	1.168 (0.796–1.713)	0.428
let-7c (high vs. low)	1.206 (0.830–1.752)	0.327
miR-26a (high vs. low)	1.110 (0.765–1.610)	0.584
miR-30d (high vs. low)	1.113 (0.769–1.610)	0.571
miR-98 (high vs. low)	1.086 (0.718–1.641)	0.696
miR-195 (high vs. low)	1.086 (0.751–1.571)	0.662
miR-202 (high vs. low)	1.455 (1.000–2.118)	0.048 *
**Multivariate Analysis**		
Cox Regression	HR (95% CI)	*p* Value
Gender (male vs. female)	2.232 (1.262–3.946)	0.006 *
No. of Metastatic Sites (≥3 vs. 0–2)	1.537 (0.924–2.556)	0.097
Liver Metastases (yes vs. no)	1.877 (1.139–3.094)	0.014 *
miR-202 (high vs. low)	1.564 (1.068–2.289)	0.021 *

HR, hazard ratio; CI, confidence intervals; ECOG PS, Eastern Cooperative Oncology Group Performance Status; patients were classified into high and low expression groups according to the median value of each miRNA; Cox regression, * *p* < 0.05.

**Table 4:** Univariate and multivariate Cox regression analysis for overall survival (OS) in NSCLC patients (N = 125).

	**Univariate Analysis**	
Cox Regression	HR (95% CI)	*p* Value
Age (<65 vs. ≥65)	1.214 (0.846–1.742)	0.292
Gender (male vs. female)	1.546 (0.933–2.622)	0.090
ECOG PS (≥2 vs. 0–1)	2.289 (1.353–3.871)	0.002 *
Stage at Diagnosis (IV vs. <IV)	1.542 (0.627–3.793)	0.346
Histology (SqCC vs. non-SqCC)	1.262 (0.850–1.874)	0.249
No. of Metastatic Sites (≥3 vs. 0–2)	1.589 (1.028–2.456)	0.037 *
Brain Metastases (yes vs. no)	1.082 (0.653–1.793)	0.759
Liver Metastases (yes vs. no)	1.826 (1.190–2.802)	0.006 *
Bone Metastases (yes vs. no)	1.416 (0.965–2.078)	0.075
let-7c (high vs. low)	1.050 (0.724–1.524)	0.797
miR-26a (high vs. low)	1.329 (0.907–1.946)	0.145
miR-30d (high vs. low)	1.251 (0.865–1.809)	0.235
miR-98 (high vs. low)	1.101 (0.725–1.673)	0.651
miR-195 (high vs. low)	1.307 (0.894–1.911)	0.167
miR-202 (high vs. low)	1.596 (1.074–2.292)	0.020 *
**Multivariate Analysis**		
Cox Regression	HR (95% CI)	*p* Value
ECOG PS (≥2 vs. 0–1)	2.065 (1.215–3.581)	0.008 *
No. of Metastatic Sites (≥3 vs. 0–2)	1.230 (0.751–2.016)	0.410
Liver Metastases (yes vs. no)	1.666 (1.033–2.687)	0.036 *
miR-202 (high vs. low)	1.558 (1.060–2.291)	0.024 *

HR, hazard ratio; CI, confidence intervals; ECOG PS, Eastern Cooperative Oncology Group Performance Status; patients were classified into high and low expression groups according to the median value of each miRNA; Cox regression, * *p* < 0.05.

## Data Availability

Data will be available upon request.

## Supplementary Materials

The following are available online at https://www.mdpi.com/article/10.3390/cells10081988/s1. Table S1: Assay ID for each miRNA used in the study. Figure S1: U6 snRNA expression levels between NSCLC patients and healthy donors. Figure S2: Expression of miR-26a associated with (a) the numbers of metastases (metastatic sites > 3 vs. 0–2) and (b) brain metastases (yes vs. no) in NSCLC patients (N = 125) treated with first-line platinum-based chemotherapy. Figure S3: Kaplan–Meier analysis for PFS (left column) and OS (right column) (from top to bottom): miR-26a, let-7c, miR-30d, miR-195 and miR-98, based on the microRNAs’ expression levels in the plasma of NSCLC patients (N = 125). Figure S4: Kaplan–Meier analysis for PFS in SqCC (left column) and non-SqCC (right column) (from top to bottom): miR-26a, let-7c, miR-30d, miR-195 and miR-98, based on the microRNAs’ expression levels in the plasma of SqCC (N–40) and non-SqCC (N = 85) NSCLC patients. Figure S5: Kaplan–Meier analysis for OS in SqCC (left column) and non-SqCC (right column) (from top to bottom): let-7c, miR-30d, miR-195 and miR-98, based on the microRNAs’ expression levels in the plasma of SqCC (N-40) and non-SqCC (N = 85) NSCLC patients. Table S2: Median Ct values, SD and median miRNA expression values in the plasma of NSCLC patients (N = 125) and healthy volunteers (N = 33). Table S3: List of macrophage related miRNAs and their statistical significance in lung cancer based on KM plotter dataset. Table S4: Univariate and multivariate Cox regression analysis for overall survival (OS) in Squamous subtype NSCLC patients (N = 40). Table S5: Univariate and multivariate Cox regression analysis for overall survival (OS) in Non-Squamous subtype NSCLC patients (N = 85). Figure S6: Survival analysis of hsa-let-7c, hsa-miR-26a, hsa-miR-30d, hsa-miR-98, hsa-miR-195 and hsa-miR-202 in (A) adenocarcinoma (N = 513) and (B) lung squamous cell carcinoma (N = 478) (KM plotter dataset).

## Abbreviations

Abbreviation	Definition
APCs	Antigen-presenting cells
cDNA	Complementary DNA
CDDP	Cis-diamminedichloridplatinum, cisplatin
CI	Confidence intervals
CT	Computed tomogaphy
Ct	Cycle threshold
CTLs	Cytotoxic T lymphocytes
ECOG	Eastern Cooperative Oncology Group
EDTA	Ethylenediamenetetraacetic acid
Et-OH	Ethanol
GALNT7	N-acetylgalactosaninyltransferase 7
GEM	Gemcitabine
HR	Hazard ratio
MATN2	Matrilin 2
miRNAs	microRNAs
MRI	Magnetic resonance imaging
M-CSF	Macrophage colony-stimulating factor
non-SqCC	non-Squamous
OS	Overall survival
PD	Progression disease
PEM	pemetrexed
PFS	Progression free survival
PR	Partial response
PS	Performance status
RT-qPCR	Real-time quantative polymerase chain reaction
SD	Stable disease
SqCC	Squamous
STAT3	Signal transducer and activator of transcription 3
TAMs	Tumor associated macrophages
TME	Tumor microenvironment
TXT	Taxotere
