# Supplementary material for: A Prognostic Role for Circulating microRNAs Involved in Macrophage Polarization in Advanced Non-Small Cell Lung Cancer
Source: Cells. 2021 Aug 5;10(8):1988. doi: 10.3390/cells10081988 (PMC8391493; doi:10.3390/cells10081988)
Supplement: Supplementary file 1 [file cells-10-01988-s001.zip › cells-1303503-supplementary.pdf]

Supplementary

Table S1. Assay ID for each miRNA used in the study

Name	Assay ID
hsa-let-7c-5p	000379
hsa-miR-26a-5p	000405
hsa-miR-30d-5p	000420
hsa-miR-98-5p	000577
hsa-miR-195-5p	000494
hsa-miR-202-3p	002363
U6 snRNA	001973
Cel-miR-39	000200

Table S2. Median Ct values, SD and median miRNA expression values in the plasma of NSCLC patients (N=125) and healthy volunteers (N=33).

	NSCLC patients (N=125)			Healthy Volunteers (N=33)	
	**Ct**	**SD**	**Median**	**Ct**	**SD**
**let-7c**	32.65	± 2.37	1.55	31.56	± 1.27
**miR-26a**	26.8	± 3.41	1.61	27.75	± 0.84
**miR-30d**	27.59	± 2.21	0.7	26.83	± 1.07
**miR-98**	34.30	± 2.50	1.16	33.73	± 1.14
**miR-195**	31.15	± 3.39	0.06	26.84	± 1.72
**miR-202**	34.30	± 1.65	1.55	34.39	± 1.55

Ct, cycle threshold; SD, standard deviation; miRNA expression values were calculated by the $2^{-\Delta\Delta Ct}$ method.

Table S3. List of macrophage related miRNAs and their statistical significance in lung cancer based on KM plotter dataset.

	Adenocarcinoma (N=513)		Lung Squamous cell carcinoma (N=478)		Reference
hsa-miR ID	p value	HR (95% CI)	p value	HR (95% CI)	DOI
let-7c	0.0014*	0.59 (0.43-0.82)	0.031*	0.73 (0.54-0.97)	10.4049/jimmunol.1202496
miR-9	0.0091*	1.48 (1.1-1.98)	0,21	1.22 (0.89-1.67)	10.1016/j.it.2013.02.003
miR-19a	0,19	0.82 (0.61-1.1)	0.026*	0.73 (0.55-0.96)	10.1038/onc.2013.258
miR-21	0.0028*	1.59 (1.17-2.15)	0,16	1.22 (0.92-1.62)	10.1016/j.it.2013.02.003
miR-26a	0.0038*	0.63 (0.45-0.86)	0.033*	0.74 (0.56-0.98)	10.1371/journal.ppat.1006410
miR-27a	0.025*	1.39 (1.04-1.87)	0,37	1.14 (0.86-1.5)	10.1074/jbc.M115.694133
miR-29	0.019*	0.69 (0.51-0.94)	0,29	0.85 (0.63-1.15)	10.3390/cells8101140
miR-30d	0.00056*	0.6 (0.45-0.8)	0,26	0.85 (0.64-1.13)	10.1016/j.ccr.2011.05.027
miR-98	0,19	0.82 (0.61-1.11)	1.3E-05**	0.54 (0.4-0.71)	10.1016/j.biochi.2018.04.016
miR-100	0,14	0.8 (0.6-1.08)	0,057	1.32 (0.99-1.75)	10.1038/s41389-018-0106-y
miR-103a	0,1	0.77 (0.57-1.05)	0.01*	0.64 (0.46-0.9)	10.1016/j.ymthe.2017.11.016
miR-124	0,41	0.88 (0.65-1.2)	0.0095*	0.69 (0.52-0.92)	10.1038/cr.2013.116
miR-125a	0.018*	0.7 (0.52-0.94)	0,076	1.3 (0.97-1.73)	10.1016/j.it.2013.02.003
miR-125b	0.04*	0.73 (0.54-0.99)	0,11	1.26 (0.95-1.68)	10.1021/acs.nanolett.8b00689
miR-127	0,21	1.21 (0.89-1.65)	0,29	0.86 (0.65-1.14)	10.4049/jimmunol.1402088
miR-130a	0,066	0.73 (0.53-1.02)	0.034*	1.4 (1.02-1.92)	10.3892/or.2015.4301
miR-145	0.02*	0.71 (0.53-0.95)	0,17	0.82 (0.62-1.09)	10.3390/cells8101140
miR-146	0.012*	0.69 (0.51-0.92)	0,06	0.76 (0.57-1.01)	10.1016/j.it.2013.02.003
miR-147	0,31	0.86 (0.63-1.16)	0.0027*	0.65 (0.49-0.86)	10.1016/j.it.2013.02.003
miR-155	0,08	0.77 (0.57-1.03)	0.0088*	0.68 (0.51-0.91)	10.1016/j.it.2013.02.003
miR-181a	0.0079*	0.66 (0.48-0.9)	0,14	1.24 (0.93-1.66)	10.1038/mtna.2016.71
miR-181b	0,1	1.3 (0.95-1.78)	0,27	0.85 (0.64-1.13)	10.1007/s12035-016-0163-1
miR-181d	0.0063*	0.67 (0.5-0.89)	0.045*	0.75 (0.57-0.99)	10.1016/j.canlet.2018.08.001
mir-187	0.0013*	0.61 (0.45-0.83)	5.8E-05**	0.5 (0.36-0.71)	10.1016/j.it.2013.02.003
miR-195	0.014*	0.69 (0.52-0.93)	0.042*	0.71 (0.51-0.99)	10.1186/s13045-019-0708-7
miR-199a	0.0099*	0.68 (0.51-0.91)	0,14	0.8 (0.6-1.07)	10.1016/j.ymthe.2019.05.019
miR-202	0.012*	0.62 (0.43-0.91)	0.021*	0.72 (0.54-0.95)	10.1159/000493803
miR-222	0,3	1.17 (0.87-1.58)	0.067*	0.77 (0.58-1.02)	10.18632/oncotarget.9246
miR-223	0,25	0.85 (0.63-1.13)	0,24	0.85 (0.64-1.12)	10.1016/j.it.2013.02.003
miR-301a	0.027*	0.7 (0.5-0.96)	0,051	0.75 (0.57-1)	10.1158/0008-5472.CAN-17-3841
miR-320a	0,28	1.2 (0.86-1.67)	0.0036*	0.63 (0.47-0.86)	10.1002/ijc.31988

miR-378	0,068	1.38 (0.97-1.96)	0.014*	0.68 (0.5-0.93)	10.1016/j.it.2013.02.003
miR-511	0,083	0.77 (0.57-1.04)	0,11	1.28 (0.95-1.74)	10.1016/j.it.2013.02.003
miR-935	0,33	1.15 (0.86-1.55)	0.013*	1.43 (1.08-1.9)	10.1007/s00262-018-2261-6
miR-1207	0,34	0.86 (0.64-1.17)	0.0027*	0.65 (0.49-0.86)	10.18632/oncotarget.8718

HR, Hazard Ratio; CI, Confidence Intervals; * $p < 0.05$; ** $p < 0.001$

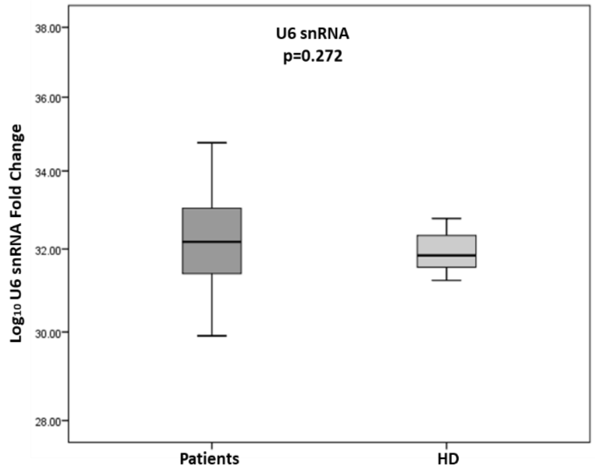

Figure S1. U6 snRNA expression levels between NSCLC patients and healthy donors. Mann-Whitney test was used to determine statistically significant differences and the results were displayed on box plots. Horizontal line depicts the median Ct value, whereas the length of the boxes is the interquartile range that represents values between the 75th and 25th percentiles of individual Ct values. P values are shown.

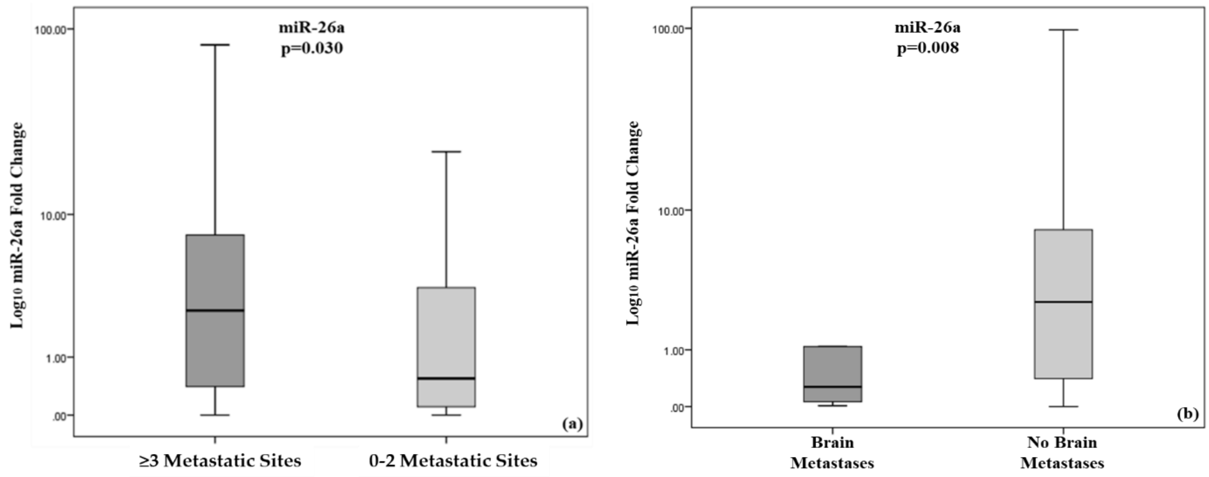

Figure S2. Expression of miR-26a associated with (a) number of metastases (metastatic sites >3 vs 0-2) and (b) brain metastases (yes vs no) in NSCLC patients (N=125) treated with first-line platinum-based chemotherapy. Expression levels of miR-26a were assessed by the $2^{-\Delta\Delta Ct}$ method and U6 snRNA was used as a reference gene. Statistically significant differences were determined by the Mann-Whitney test and the results are displayed on box plots. Horizontal line depicts median, whereas the length of the boxes is the interquartile range that represents values between the 75th and 25th percentiles of individual fold change expression values. Relative expression values on the *y*-axis are plotted on a $\log_{10}$ scale. *p* values are shown.

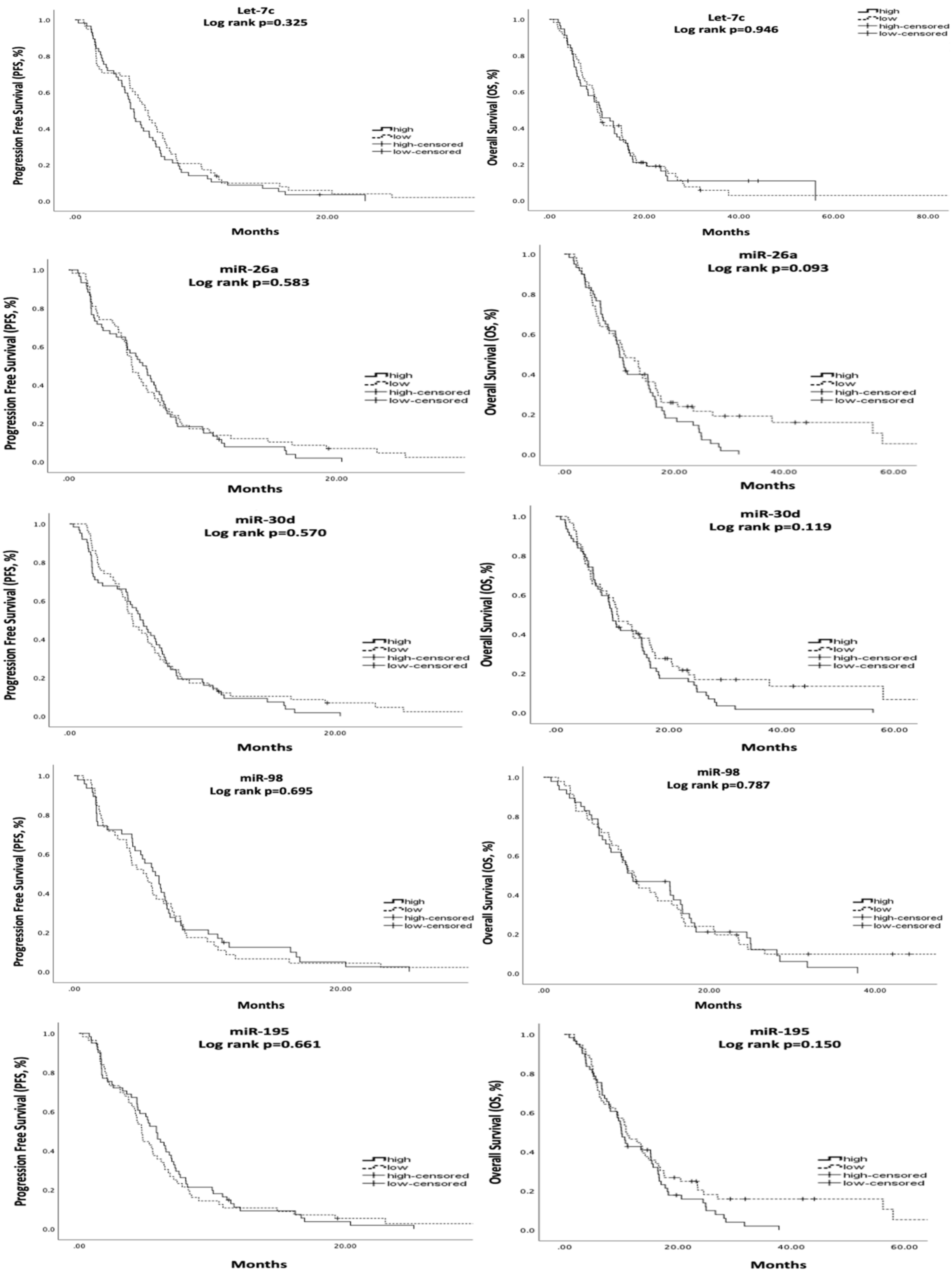

Figure S3. Kaplan-Meier analysis for PFS (left column) and OS (right column) (from top to bottom): let-7c, miR-26a, miR-30d, miR-98 and miR-195, based on the microRNA's expression levels in the plasma of NSCLC patients (N=125). Median expression values for each microRNA subcategorized patients into high and low expression groups. Curves were compared using the log rank test. *p* values are shown.

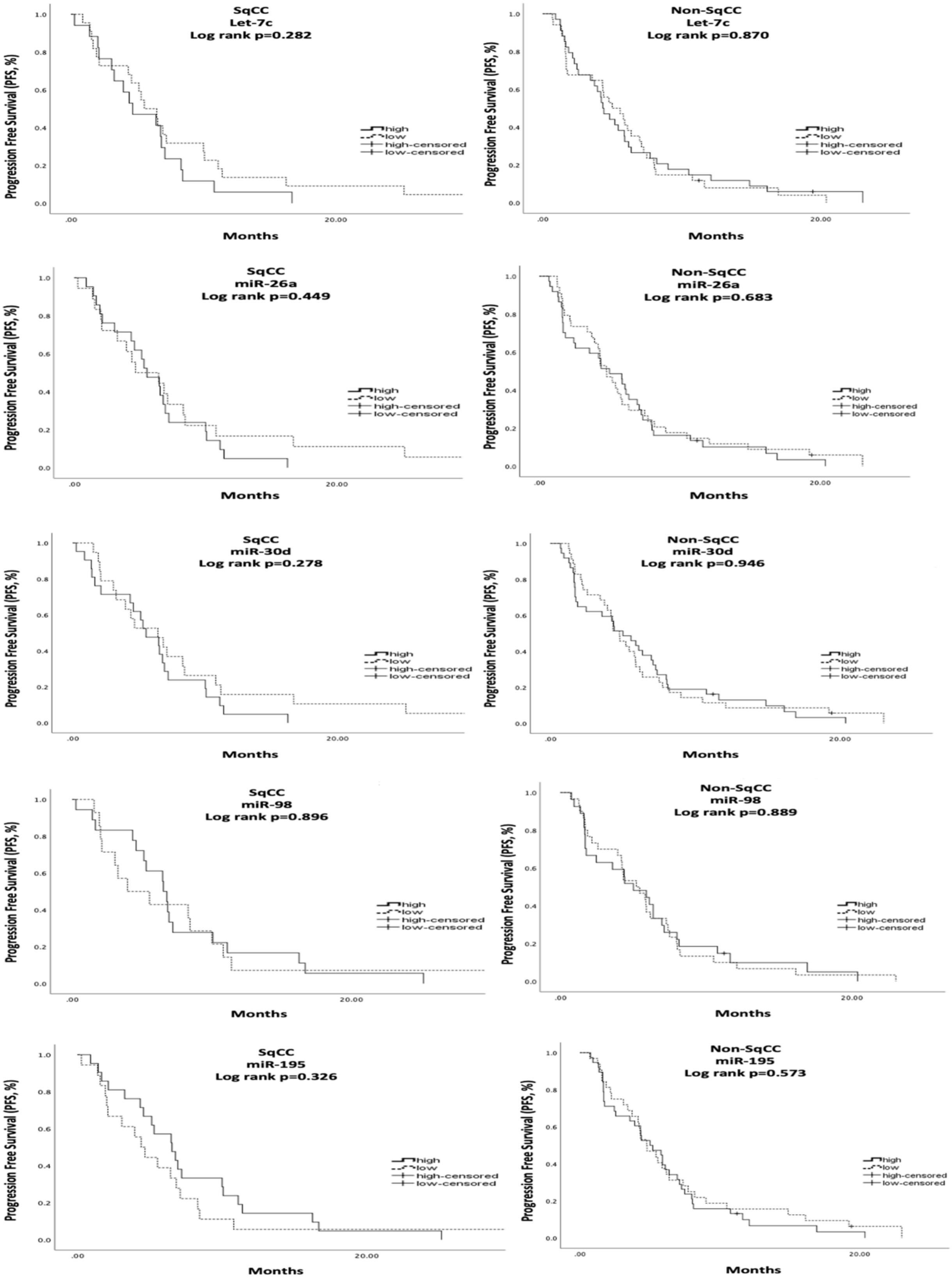

Figure S4. Kaplan-Meier analysis for PFS in SqCC (left column) and Non-SqCC (right column) (from top to bottom): let-7c, miR-26a, miR-30d, miR-98 and miR-195, based on the microRNA's expression levels in the plasma of SqCC (N-40) and Non-SqCC (N=85) NSCLC patients. Median expression values for each microRNA subcategorized patients into high and low expression groups. Curves were compared using the log rank test. p values are shown.

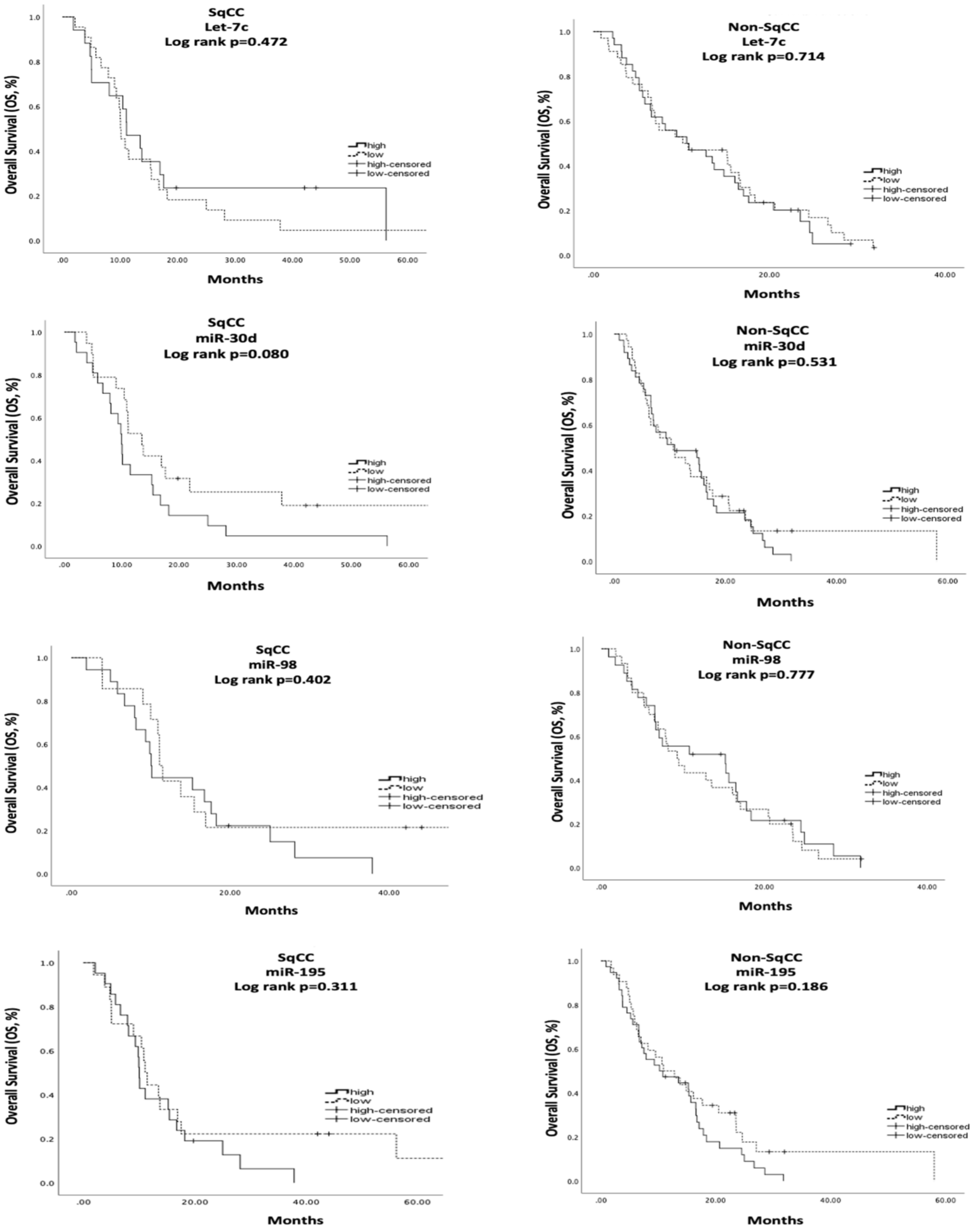

Figure S5. Kaplan-Meier analysis for OS in SqCC (left column) and Non-SqCC (right column) (from top to bottom): let-7c, miR-30d, miR-98 and miR-195, based on the microRNA's expression levels in the plasma of SqCC (N-40) and Non-SqCC (N=85) NSCLC patients. Median expression values for each microRNA subcategorized patients into high and low expression groups. Curves were compared using the log rank test. *p* values are shown.

Table S4. Univariate and Multivariate Cox regression analysis for overall survival (OS) in Squamous subtype NSCLC patients (N=40).

**Univariate Analysis**		
Cox Regression	HR (95% CI)	*p* Value
Age (<65 vs. ≥65)	1.634 (0.844-3.164)	0.145
ECOG PS (≥2 vs. 0-1)	2.401 (1.007-5.725)	0.048*
Stage at Diagnosis (IV vs. <IV)	1.377 (0.535-3.547)	0.507
No. of Metastatic Sites (≥3 vs. 0-2)	1.421 (0.487-4.149)	0.520
Liver Metastases (yes vs. no)	1.920 (0.869-4.242)	0.107
Bone Metastases (yes vs. no)	2.170 (0.911-5.166)	0.080
let-7c (high vs. low)	1.117 (0.585-2.133)	0.736
miR-26a (high vs. low)	2.054 (1.010-4.197)	0.047*
miR-30d (high vs. low)	1.567 (0.828-2.965)	0.168
miR-98 (high vs. low)	1.462 (0.683-3.126)	0.328
miR-195 (high vs. low)	1.500 (0.757-2.974)	0.246
miR-202 (high vs. low)	1.231 (0.629-2.412)	0.544
**Multivariate Analysis**		
Cox Regression	HR (95% CI)	p Value
ECOG PS (≥2 vs. 0-1)	1.941 (0.947-3.977)	0.070
miR-26a (high vs. low)	2.063 (0.861-4.944)	0.104

HR, Hazard Ratio; CI, Confidence Intervals; ECOG PS, Eastern Cooperative Oncology Group Performance Status; patients were classified into high and low expression groups according to the median value of each miRNA; Cox regression, * $p < 0.05$, all patients in the SqCC group did not have brain metastases.

Table S5. Univariate and Multivariate Cox regression analysis for overall survival (OS) in Non-Squamous subtype NSCLC patients (N=85).

Univariate Analysis		
Cox Regression	HR (95% CI)	*p* Value
Age (<65 vs. ≥65)	1.013 (0.641-1.602)	0.954
Gender (male vs. female)	1.416 (0.810-2.473)	0.222
ECOG PS (≥2 vs. 0-1)	2.321 (1.100-4.896)	0.027*
No. of Metastatic Sites (≥3 vs. 0-2)	1.740 (1.023-2.960)	0.041*
Brain Metastases (yes vs. no)	1.204 (0.653-2.220)	0.553
Liver Metastases (yes vs. no)	1.855 (1.082-3.182)	0.025*
Bone Metastases (yes vs. no)	1.147 (0.712-1.846)	0.573
let-7c (high vs. low)	1.185 (0.721-1.947)	0.502
miR-26a (high vs. low)	1.116 (0.689-1.810)	0.655
miR-30d (high vs. low)	1.103 (0.683-1.780)	0.689
miR-98 (high vs. low)	1.031 (0.606-1.754)	0.911
miR-195 (high vs. low)	1.334 (0.817-2.178)	0.250
miR-202 (high vs. low)	1.978 (1.198-3.267)	0.008*
**Multivariate Analysis**		
Cox Regression	HR (95% CI)	*p* Value
ECOG PS (≥2 vs. 0-1)	2.171 (0.974-4.837)	0.058
No. of Metastatic Sites (≥3 vs. 0-2)	1.413 (0.743-2.687)	0.291
Liver Metastases (yes vs. no)	1.368 (0.697-2.686)	0.362
miR-202 (high vs. low)	1.989 (1.196-3.309)	0.008*

HR, Hazard Ratio; CI, Confidence Intervals; ECOG PS, Eastern Cooperative Oncology Group Performance Status; patients classified into high and low expression groups according to the median value of each miRNA; Cox regression, * $p < 0.05$, all patients in the non-SqCC group were stage IV.

(A)

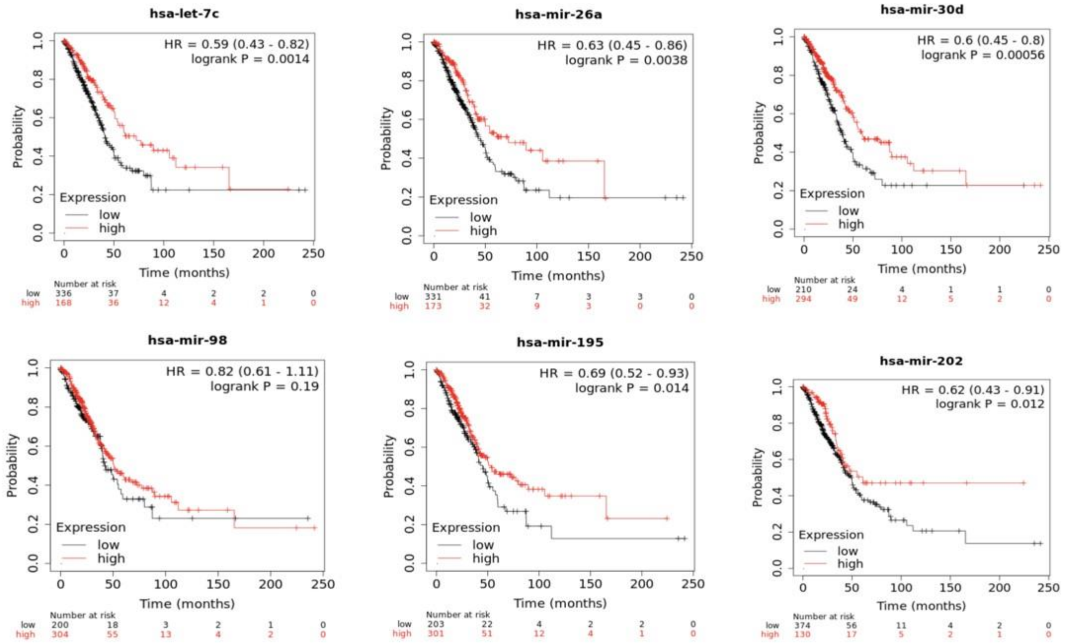

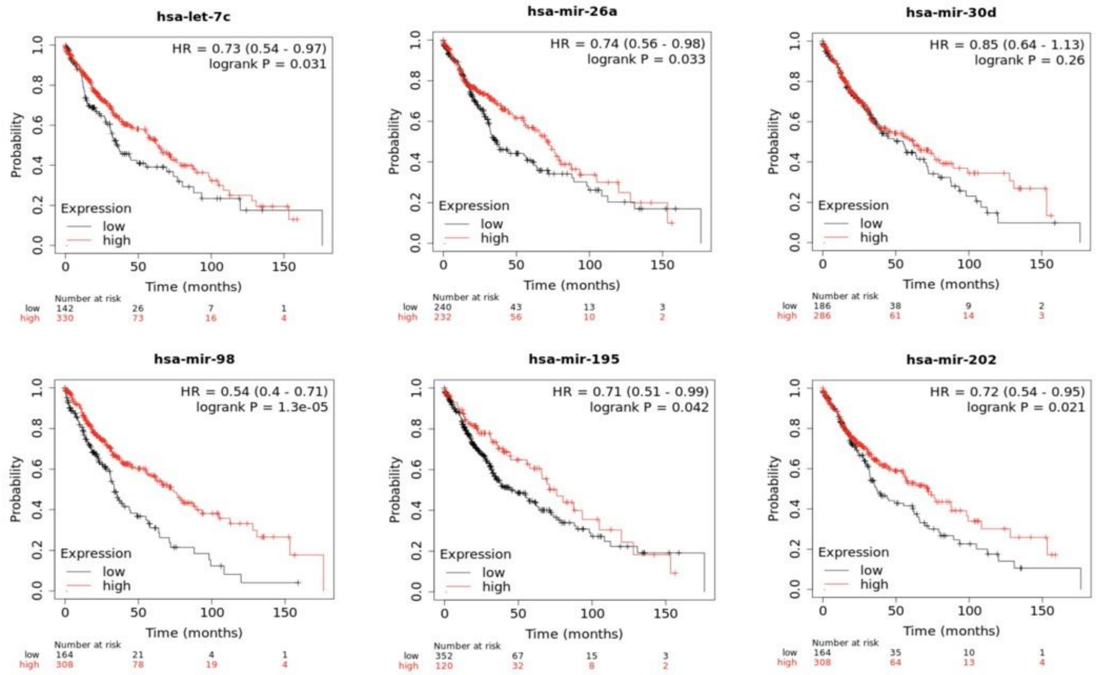

(B)

Figure S6. Survival analysis of hsa-let-7c, hsa-miR-26a, hsa-miR-30d, hsa-miR-98, hsa-miR-195 and hsa-miR-202 in (A) Adenocarcinoma (N=513) and (B) Lung Squamous cell carcinoma (N=478), (KM plotter dataset). Samples are categorized as high (red) and low (black) expression groups for each miRNA. Hazard ratio (HR) and p value for each miRNA associated with survival are shown within the respective plot.
